# Cryptosporidium and Toxoplasma Parasites Are Inhibited by a Benzoxaborole Targeting Leucyl-tRNA Synthetase

**DOI:** 10.1128/AAC.00873-16

**Published:** 2016-09-23

**Authors:** Andrés Palencia, Ru-Juan Liu, Maria Lukarska, Jiri Gut, Alexandre Bougdour, Bastien Touquet, En-Duo Wang, Xianfeng Li, M. R. K. Alley, Yvonne R. Freund, Philip J. Rosenthal, Mohamed-Ali Hakimi, Stephen Cusack

**Affiliations:** a European Molecular Biology Laboratory, Grenoble Outstation, Grenoble, France, and University Grenoble Alpes-CNRS-EMBL International Unit (UMI 3265) for Virus Host-Cell Interactions, UMI 3265, Grenoble, France; b Institute for Advanced Biosciences, Team Host-Pathogen Interactions & Immunity to Infection, INSERM U1209, CNRS UMR5309, Université Grenoble Alpes, Grenoble, France; c Institute of Biochemistry and Cell Biology, Chinese Academy of Sciences, Shanghai, People's Republic of China; d Department of Medicine, University of California, San Francisco, California, USA; e Anacor Pharmaceuticals, Palo Alto, California, USA

## Abstract

The apicomplexan parasites Cryptosporidium and Toxoplasma are serious threats to human health. Cryptosporidiosis is a severe diarrheal disease in malnourished children and immunocompromised individuals, with the only FDA-approved drug treatment currently being nitazoxanide. The existing therapies for toxoplasmosis, an important pathology in immunocompromised individuals and pregnant women, also have serious limitations. With the aim of developing alternative therapeutic options to address these health problems, we tested a number of benzoxaboroles, boron-containing compounds shown to be active against various infectious agents, for inhibition of the growth of Cryptosporidium parasites in mammalian cells. A 3-aminomethyl benzoxaborole, AN6426, with activity in the micromolar range and with activity comparable to that of nitazoxanide, was identified and further characterized using biophysical measurements of affinity and crystal structures of complexes with the editing domain of Cryptosporidium leucyl-tRNA synthetase (LeuRS). The same compound was shown to be active against Toxoplasma parasites, with the activity being enhanced in the presence of norvaline, an amino acid that can be mischarged by LeuRS. Our observations are consistent with AN6426 inhibiting protein synthesis in both Cryptosporidium and Toxoplasma by forming a covalent adduct with tRNA^Leu^ in the LeuRS editing active site and suggest that further exploitation of the benzoxaborole scaffold is a valid strategy to develop novel, much needed antiparasitic agents.

## INTRODUCTION

*C*ryptosporidium and Toxoplasma are important protozoan pathogens of humans. Cryptosporidium species cause acute gastrointestinal infections in young children, leading to high morbidity in developing countries (1–4). About 15 different species have been identified in human infections, although Cryptosporidium parvum and Cryptosporidium hominis are the primary pathogens (1). In a recent large study at seven sites in Asia and Africa, Cryptosporidium was the second most common attributable cause of moderate-to-severe diarrhea in children under 2 years of age (2), a disease that, according to a WHO 2016 report, causes 18% of the deaths in children under 5 years. Cryptosporidium also causes serious outbreaks due to the resistance of the parasite to chlorine or other disinfectants (3). An outbreak in Milwaukee, WI, in 1993 affected almost half a million people (5). Additionally, it can be a life-threatening pathogen in immunocompromised individuals, especially those with advanced AIDS (1). Nitazoxanide, the drug recommended for the treatment of cryptosporidiosis, has very limited efficacy in both malnourished and immunocompromised individuals (6). Toxoplasma gondii infects animals and humans, with about 30% of people carrying the parasite, mostly in its latent form (7, 8). T. gondii is a common cause of serious congenital infections, resulting in both severe fetal disease and subsequent illnesses in children and adults. Toxoplasmosis also causes serious illnesses in immunocompromised individuals, including encephalitis, chorioretinitis, pneumonitis, and myocarditis (9, 10). For the drugs currently used to treat toxoplasmosis, long courses of therapy are required, and their use is often limited by side effects (11, 12). Importantly, there is no available drug with efficacy against the slowly growing bradyzoite stage of the parasite, which forms tissue cysts in deep organs, such as the brain, and can reactivate years after initial infection (13).

Despite the seriousness of cryptosporidiosis and toxoplasmosis, interest in the development of new drugs targeting these pathogens has been limited (14), as evidenced by the absence of new drugs in clinical trials (www.clinicaltrials.gov). To identify new molecules with activity against Cryptosporidium, we tested several benzoxaborole compounds, building on the known activity against fungal and bacterial aminoacyl-tRNA synthetases (AARSs) of some compounds of this class (15, 16). AARSs play an essential role in protein synthesis by charging tRNAs with their cognate amino acids (17). Despite the fact that homologous proteins exist in humans, AARSs are good targets for antimicrobial drug design, since structural and sequence divergences can be exploited to enhance specificity and avoid toxicity (18, 19). Indeed, AARSs are validated drug targets, with several AARS inhibitors demonstrating medical applications (reviewed in references 18, 20, and 21).

The discovery of tavaborole, a small benzoxaborole approved for use for the topical treatment of onychomycosis (22), as a fungal leucyl-tRNA synthetase (LeuRS) inhibitor (16) led to the development of 3-aminomethyl derivatives that are effective *in vivo* against Gram-negative bacteria (15). Here, we present evidence, based on cell-based models of infection, biophysics, and X-ray crystallography, that a 3-aminomethyl benzoxaborole (AN6426) is active against C. parvum and T. gondii through inhibition of LeuRS and that further exploitation of this novel mechanism of action could lead to new antiparasitic agents.

## MATERIALS AND METHODS

### Fluorescence microscopy assays to test activity against C. parvum in MDCK cells.

The compounds (*S*)-3-(aminomethyl)-4-chloro-7-ethoxybenzo[*c*][1,2]oxaborol-1(3*H*)-ol (AN6426) and (*S*)-3-(aminomethyl)-4-bromo-7-ethoxybenzo[*c*][1,2]oxaborol-1(3*H*)-ol (AN8432) were synthesized as described elsewhere (23, 24). Nitazoxanide (Sigma) was used as a positive control. Activity against C. parvum was monitored as previously described (25), with modifications. Madin-Darby canine kidney (MDCK) type 2 (ATCC CRL-2936) cells were grown at 37°C under 5% CO_2_ in optical-quality 384-well flat-bottom plates to confluence in Dulbecco's minimum essential medium (DMEM; Life Technologies) supplemented with 5% fetal calf serum (FCS; Life Technologies). Before addition of parasites, the concentration of FCS in the medium was reduced to 1%. Oocysts of C. parvum strain Iowa were purchased from the Sterling Parasitology Laboratory at the University of Arizona (http://microvet.arizona.edu/research/crypto/) and stored at 4°C until use. Immediately before addition to the MDCK cells, oocysts were sequentially incubated at 37°C for 10 min in 10 mM HCl and for 10 min at 15°C in 2 mM sodium taurocholate to prime them for excystation. Treated oocysts were then added to a confluent monolayer of host cells in optical-quality 384-well plates and incubated with the test compounds over a concentration range 4.6 nM to 10 μM at 37°C under 5% CO_2_ for 48 h. After incubation, the cells were fixed with 4% buffered formaldehyde, extracted, and blocked with 0.1% Triton X-100, 0.25% bovine serum albumin (BSA) in saline. Parasites were identified by staining with biotinylated Vicia villosa lectin (VVL; 0.5 μg/ml; Vector Laboratories), washing, and then staining with 0.5 μg/ml Cy3-streptavidin (Jackson ImmunoResearch) and 0.5 μg/ml 2-(4-amidinophenyl)-1*H*-indole-6-carboxamidine (DAPI). Parasites and host cells were imaged with a GE InCell 2000 automated microscope, with epifluorescence images of host cell nuclei stained with DAPI and intracellular parasites stained with Cy3 being obtained. GE InCell Developer (version 1.9) image analysis software was used to quantify the parasites and host cells in each image. The Cryptosporidium count was divided by the host cell nucleus count to provide the number of parasites per cell. The 50% effective concentrations (EC_50_s) were calculated from two independent experiments (one performed in duplicate and one performed in triplicate) from nonlinear regression curves using the XE program, and curves were plotted with the Spotfire program.

### Production of cytosolic editing domain of *Cm*LeuRS.

A DNA fragment encoding cytosolic C. muris LeuRS (*Cm*LeuRS) connective polypeptide domain (CP1) residues 254 to 541 was cloned into the NcoI-XhoI sites of pETM-11 (EMBL). The resulting construct contained an N-terminal hexahistidine tag and a tobacco etch virus (TEV) cleavage site at the *Cm*LeuRS-coding sequence, resulting in addition of a GAMG sequence at the N terminus after TEV cleavage. Proteins were expressed in the Rosetta 2(DE3) or BL21-Codon+RIL strain at 16°C. The cells were lysed by sonication in lysis buffer (20 mM Tris-HCl, pH 7.5, 150 mM NaCl, 5 mM β-mercaptoethanol, 10 mM imidazole, 1 mM protease inhibitors). The protein from the soluble fraction was loaded onto a nickel column (Ni-nitrilotriacetic acid; Qiagen), which was washed with (i) 50 ml of lysis buffer plus 50 mM imidazole, (ii) 50 ml of lysis buffer plus 1 M NaCl, and (iii) 50 ml of lysis buffer. Proteins were eluted from the column with 15 ml of lysis buffer plus 200 to 400 mM imidazole. The His tag was cleaved with TEV protease by dialysis in buffer containing 20 mM Tris-HCl, pH 7.5, 100 mM NaCl, and 7 mM β-mercaptoethanol at 4°C (18 h), and the cleaved protein was purified from the flowthrough of a second nickel column. Pure protein fractions were concentrated to a final protein concentration of about 500 μM.

### ITC experiments.

Isothermal titration calorimetry (ITC) experiments were performed at 25°C using an ITC200 system (MicroCal). Protein was dialyzed against buffer containing 50 mM HEPES-KOH, 30 mM KCl, 30 mM MgCl_2_, and 5 mM 2-β-mercaptoethanol, pH 7.5. Protein solutions were at 50 μM, and posttransfer editing analogues were at 10 mM. For the experiment with AN6426 (at 1 mM), AMP was added at 10 mM both in the sample cell and in the syringe. The heat that evolved after each ligand injection was obtained from the integral of the calorimetric signal. The resulting binding isotherms were fitted by nonlinear least squares to a single-site binding model. Analysis of the data was performed using MicroCal Origin software (version 7.0; OriginLab). Experiments were performed at least twice. The variability in the binding experiments was estimated to be 5% in the binding enthalpy and 10% in the binding affinity.

### Crystallization.

Crystallization of all *Cm*LeuRS proteins was carried out at 20°C by the hanging drop vapor diffusion method. Crystals of the apo-*Cm*LeuRS were obtained by mixing 1 μl of protein with 1 μl of reservoir solution containing 0.1 M MES (morpholineethanesulfonic acid; pH 6.8), 2% ethanol, and 10% (wt/vol) polyethylene glycol (PEG) 20000. The crystals were frozen in liquid nitrogen after transfer for a few seconds in the mother liquor, which contained 20% (vol/vol) ethylene glycol as a cryoprotectant. For the *Cm*LeuRS–2-(l-isoleucyl)amino-2-deoxyadenosine (Ile2AA) complex, solutions were prepared with 500 μM protein and 5 mM posttransfer editing analogue (Ile2AA); for the *Cm*LeuRS-AN6426-AMP complex, solutions were prepared with 500 μM protein, 1 mM AN6426, and 10 mM AMP. Crystals were obtained by mixing 1 μl of this solution with 1 μl of reservoir solution containing 1.4 M sodium/potassium phosphate, pH 5.5, and 20% glycerol was used as the cryoprotectant. For the *Cm*LeuRS–2-(l-norvalyl)amino-2-deoxyadenosine (Nva2AA) complex, solutions were prepared with 500 μM *Cm*LeuRS and 2.5 mM posttransfer editing analogue (Nva2AA). Crystals were obtained by mixing 2 μl of this solution with 2 μl of reservoir solution containing 0.1 M KNO_3_, pH 6.9, and 22% (wt/vol) PEG 3350. The crystals were frozen in liquid nitrogen after transfer in the mother liquor, which contained 20% ethylene glycol as the cryoprotectant.

### Structure determination and refinement.

The diffraction data sets of *Cm*LeuRS CP1 complex with AN6426-AMP were collected at the French National Synchrotron Facility (SOLEIL, France), the diffraction data sets of apo-*Cm*LeuRS CP1 and its complex with Nva2AA were collected at the European Synchrotron Radiation Facility (ESRF; France), and the diffraction data sets of *Cm*LeuRS CP1 complex with Ile2AA were collected at the Shanghai Synchrotron Radiation Facility (SSRF; China). The data sets were integrated and scaled with the XDS suite (26) or with the HKL2000 program package (27). Further data analysis was performed with the CCP4 suite (28). The structure of *Cm*LeuRS CP1 was initially solved by molecular replacement with the PHASER program (29) using the structure of Candida albicans LeuRS CP1 (PDB accession number 2WFG) as a model. The obtained model was improved by manual adjustments with the Coot program (30). The structure of the *Cm*LeuRS-Ile2AA complex was solved by molecular replacement with PHASER using the apo-*Cm*LeuRS CP1 as a model. All models were refined using REFMAC5 (31) and/or Phenix (32) software. Structure quality was analyzed with the PDBe validation server (http://wwpdb-validation.wwpdb.org/validservice/) and showed all residues in allowed regions for the different models. Figures were drawn with the PyMOL program (http://www.pymol.org/).

### Homology model of *Tg*LeuRS and docking of the adduct AN6426-Ade76.

The model of Toxoplasma gondii LeuRS (*Tg*LeuRS) was built by using the protein structure homology server SWISS-MODEL (33) and the structures of cytosolic apo-*Cm*LeuRS and human LeuRS (PDB accession number 2WFD) as the templates. The sequence similarity between the Toxoplasma and Cryptosporidium editing domains was 52.6%, and that between the Toxoplasma and Homo sapiens editing domains was 38.1%. Residues 1 to 312 of *Tg*LeuRS were used to build the homology model, which presents a Qmean Z score of −3.43 and a QMean4 score of −0.56, indicating a good global-quality model (34). Docking of AN6426-AMP into the Toxoplasma LeuRS's editing site was done by secondary structure matching of the model of *Tg*LeuRS described above onto the structure of the complex of *Cm*LeuRS with the adduct AN6426-AMP. No steric clashes or violations of the interatomic Van der Waals radii were found after the adduct AN6426-AMP was placed at the editing site of *Tg*LeuRS. The root mean square deviation (RMSD) calculated over the backbone atoms of *Cm*LeuRS and *Tg*LeuRS was 1.27 Å.

### Fluorescence microscopy assay to test activity against Toxoplasma in human fibroblasts.

A stock solution of AN6426 in sterilized ultrapure water was prepared at 50 mM and diluted in tissue culture medium for *in vitro* experiments. A pyrimethamine (Sigma-Aldrich) stock was prepared at 10 mM in ethanol and used as a positive control for anti-Toxoplasma activity. A norvaline (Sigma-Aldrich) stock was prepared at 100 mM in sterilized ultrapure water. All compound solutions were stored at −20°C. Type I Toxoplasma reference laboratory strain RH was used to infect a human foreskin fibroblast (HFF; primary dermal fibroblasts; normal, human, adult; ATCC PCS-201-012) monolayer under tachyzoite growth conditions in DMEM supplemented with 1% fetal bovine serum (FBS), 4 mM glutamine, 500 U/ml penicillin, and 250 μg/ml streptomycin (all from Life Technologies) at 37°C in 5% CO_2_. HFFs in 4-well plates were infected with 5 × 10^4^ extracellular tachyzoites/well. After invasion of parasites into the cells (∼8 h), the medium was replaced by medium containing either AN6426 at different concentrations (range, 12.5 to 400 μM) or pyrimethamine as the control (2 μM). For the experiments with norvaline, the final concentration was 10 mM. At each time point (every 24 h for 4 days), the slides were fixed and labeled for immunofluorescence phenotypic analysis. For immunofluorescence labeling, HFFs grown on coverslips infected with the parasites were fixed for 20 min with phosphate-buffered saline (PBS) containing 5% formaldehyde and permeabilized for 20 min with 0.2% Triton X-100. Blocking was performed with PBS containing 5% FBS and 5% goat serum for 1 h. All antibodies were diluted in 1% FBS. Coverslips were incubated for 1 h with the primary antibody and anti-small ubiquitin-like modifier (anti-SUMO), which served as a marker for T. gondii parasites (35), followed by the secondary antibodies, goat anti-mouse IgG coupled with Alexa Fluor 568 dye (Invitrogen), at a 1:1,000 dilution. The nuclei of host cells and parasites were stained for 10 min with Hoechst 33258 dye at 2 μg/ml in PBS. After 3 washes, coverslips were mounted on a glass slide with Mowiol mounting medium, and images were acquired with a fluorescence microscope (Axioplan 2; Carl Zeiss, Inc.).

### Determination of EC_50_s against T. gondii in human foreskin fibroblasts.

EC_50_s and cell viability were determined at 24 h postinfection by automatic microscope-based screening (ScanR screening station; Olympus, Germany) in 96-well plates. Infected cells were incubated with AN6426 at concentrations ranging from 12.5 to 200 μM with or without 10 mM norvaline. After 24 h, Hoechst 33342 (Life Technologies) stain was added to live cells/parasites at 5 μg/ml for 20 min to count the number of cells and parasite nuclei. Cells and parasites were fixed with prewarmed formaldehyde at 3.7% for 10 min at 37°C. Anti-GRA1 antibodies (Biotem, France) followed by Alexa Fluor 488-conjugated secondary antibodies (Molecular Probes, Life Technologies, France) were used to identify parasitophorous vacuoles (PV). A total of 20 images per well were recorded using a 20× objective. To identify parasite DNA within delimited PV boundaries and the cell nuclei, ScanR analysis was used. PV and cell nuclei were identified by the use of the intensity threshold algorithm with cutoff pixels of 250 and 400 for PV and cell nuclei, respectively, and parasite DNA detection was done with the edge segmentation tool of the ScanR careening station. Cell viability was expressed as the percentage of total cells after drug treatment with respect to the number of dimethyl sulfoxide (DMSO)-treated control cells, and parasite growth inhibition (GR) was expressed as the ratio of the total number of parasites divided by the total number of vacuoles per well. The calculated activity was normalized to the percent growth inhibition on the basis of the values obtained with the controls, which were an apparent pyrimethamine EC_100_ (effective concentration showing 100% activity, meaning the maximum measured growth inhibition ratio) and the value obtained with 0.8% DMSO (0% activity, meaning minimum growth inhibition) according to the following formula: 1 − [(measured GR − μGREC100)/(μGRDMSO − μGREC100)] × 100, where μGREC100 is maximum measured growth inhibition and μGRDMSO is minimum measured growth inhibition. EC_50_s were determined by fitting the dose-response curve using GraphPad Prism software.

### Accession numbers.

 The atomic coordinates and structure factors have been deposited in the Protein Data Bank (PDB) under the following accession numbers: 5FON for the apo-*Cm*LeuRS structure, 5FOL for the complex of *Cm*LeuRS with Ile2AA, 5FOG for the complex of *Cm*LeuRS with Nva2AA, and 5FOM for the complex of *Cm*LeuRS with the adduct AN6426-AMP.

## RESULTS

### Activity of benzoxaboroles against C. parvum.

Based on the previous interest in benzoxaboroles as therapeutics against fungi and bacteria, we tested new benzoxaboroles for activity against C. parvum-infected Madin-Darby canine kidney (MDCK) cells (25). We identified two related 3-aminomethyl benzoxaboroles, AN6426 and AN8432, to be potent inhibitors of C. parvum development, with EC_50_s of 2.2 and 6.9 μM, respectively (Table 1; see also Fig. S1 in the supplemental material). The compounds showed activity comparable to that of nitazoxanide, the current standard of care for the treatment of cryptosporidiosis (36).

**TABLE 1:**
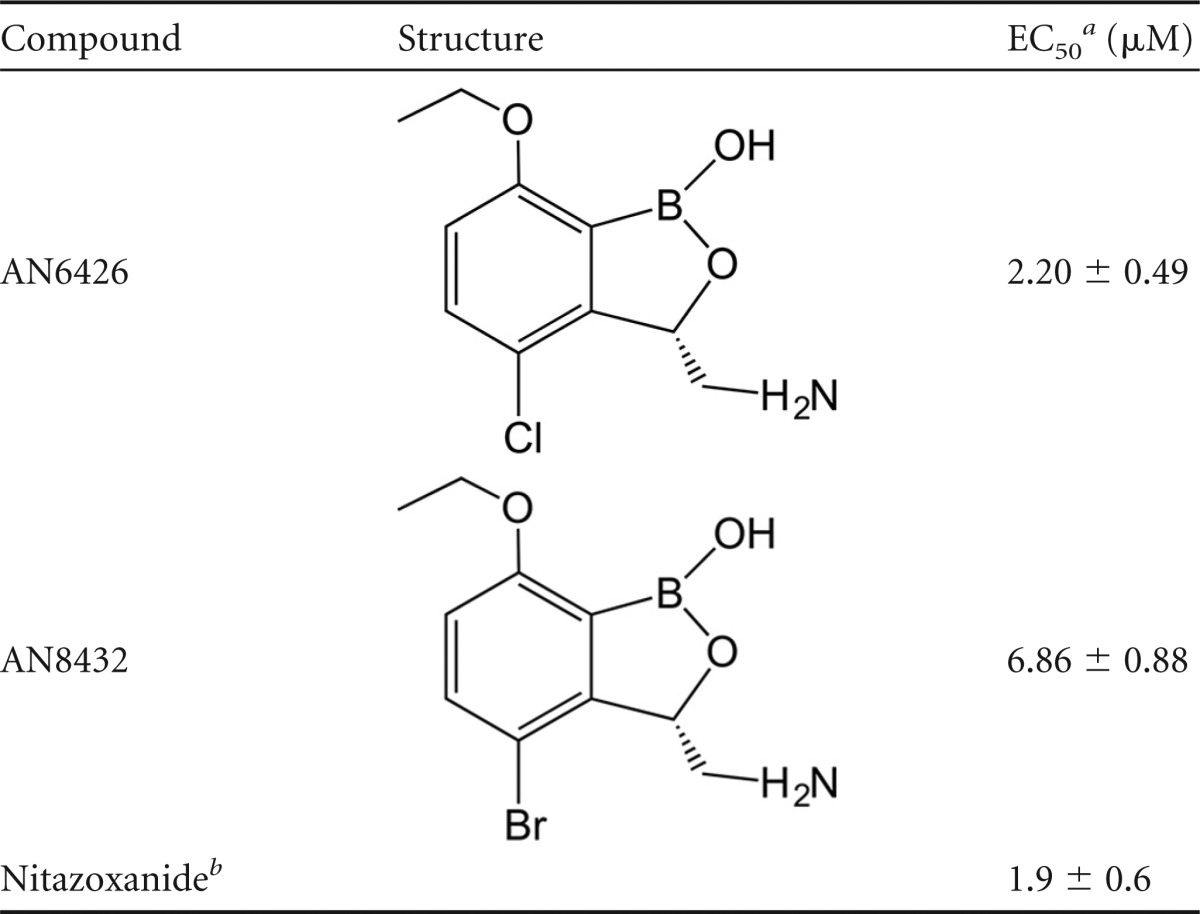
Efficacy against C. parvum in mammal cells

a EC_50_s correspond to the means from two independent experiments, one of which was performed in duplicate and one of which was performed in triplicate.

b Nitazoxanide is the standard of care for the treatment of cryptosporidiosis.

### AN6426 binds to the C. muris LeuRS editing site.

**FIG 1:**
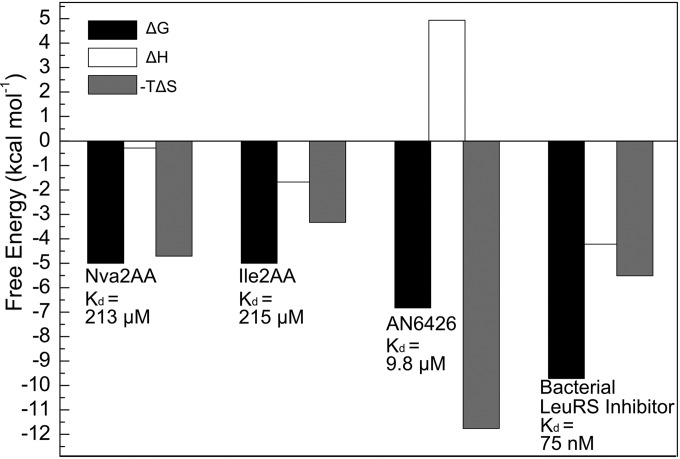
Energetics of binding of posttransfer editing analogues and AN6426-AMP to C. muris LeuRS. Gibbs free energy values (Δ*G*) calculated from the calorimetric titrations of posttransfer editing analogues (Nva2AA and Ile2AA) and AN6426-AMP into *Cm*LeuRS are shown. The affinity of the ligands (indicated as Δ*G*; *K_d_* values for each ligand are shown for reference) was dissected into the enthalpic (Δ*H*) and entropic (−*T*Δ*S*, where *T* is temperature and *S* is entropy) contributions and revealed thermodynamic contributions opposite those of bacterial LeuRS inhibitors. Results for a representative example of a Mycobacterium tuberculosis LeuRS inhibitor (PDB accession number 5AGT) are shown for reference.

Since 3-aminomethyl benzoxaboroles are known to bind to the bacterial LeuRS editing domain (15, 37), we hypothesized that AN6426 and AN8432 activity was due to the inhibition Cryptosporidium LeuRS. Therefore, we expressed the editing domain of the C. muris cytoplasmic LeuRS (*Cm*LeuRS) to enable *in vitro* biophysical and structural studies. Inspection of the genomes of several Cryptosporidium species, including the C. muris genome, suggests that there is only one cytoplasmic LeuRS gene (38–40). Similar to other LeuRSs, the C. muris LeuRS contains a predicted editing domain exhibiting highly conserved motifs that are important for the hydrolysis of tRNA^Leu^ mischarged with noncognate amino acids, such as isoleucine or norvaline, while excluding cognate Leu-tRNA^Leu^ (41, 42). We predicted the boundaries of the *Cm*LeuRS editing domain based on sequence homology with other studied eukaryotic LeuRS enzymes (43). *Cm*LeuRS and apicomplexan LeuRSs in general contain three idiosyncratic insertions at the cytoplasmic LeuRS editing domain (see Fig. S2 in the supplemental material). We suspected that the insertions, which can be highly variable, could be structurally disordered (IUPred web server values, ∼0.8 [44]). Nevertheless, we expressed and purified the complete *Cm*LeuRS editing domain. Since posttransfer editing by LeuRS in apicomplexans has not been characterized, we performed binding experiments with the *Cm*LeuRS editing domain and posttransfer editing substrate analogues for norvaline, 2-(l-norvalyl)amino-2-deoxyadenosine (Nva2AA), and isoleucine, 2-(l-isoleucyl)amino-2-deoxyadenosine (Ile2AA), which mimic a mischarged amino acid attached to Ade76. Calorimetric titrations yielded dissociation constants (*K_d_*s) of 213 and 215 μM for Nva2AA and Ile2AA, respectively (Fig. 1; see also Table S1 in the supplemental material), over the range of affinities observed for bacterial LeuRS (45), but they were lower than those seen for other editing tRNA synthetases, such as class II threonine-tRNA synthetase (ThrRS) (46). These data, together with the conservation of important active-site residues (see Fig. S2 in the supplemental material), suggest that *Cm*LeuRS preserves posttransfer editing activity.

Next, we performed binding assays using AN6426 and AMP, which together can form an AN6426-AMP adduct that mimics the charged Ade76 of the tRNA acceptor stem. We measured a *K_d_* of 9.8 μM for AN6426-AMP (Fig. 1), indicating a 20-fold increase of affinity compared to that of the posttransfer editing substrate analogues Nva2AA and Ile2AA. In addition, calorimetry showed that the interaction is entropically driven, a binding signature that contrasts with that of the Nva2AA and Ile2AA analogues and editing site inhibitors binding to bacterial LeuRS, which present favorable binding enthalpies (Fig. 1). The favorable entropy measured for AN6426 binding to *Cm*LeuRS suggests that hydrophobic contacts are more important at the *Cm*LeuRS editing site than the bacterial LeuRS editing site (Fig. 1). A favorable entropy might also be associated with the release of solvent molecules at the binding interface upon complex formation (for a review, see references 47 and 48).

### Structural insights on posttransfer editing by *Cm*LeuRS.

To investigate structural details of posttransfer editing by *Cm*LeuRS and to gain insight into the mode of binding of AN6426, we determined high-resolution structures of the apo *Cm*LeuRS editing domain, *Cm*LeuRS in complexes with the posttransfer editing analogues Nva2AA and Ile2AA, and *Cm*LeuRS in complex with AN6426-AMP bound to the editing site (Table 2; see also Fig. S3 and S4 in the supplemental material for a summary).

**TABLE 2:** Data collection and refinement statistics of crystal structures

Parameter^*a*^	Values(s) for^*b*^:			
	Apo-*Cm*LeuRS CP1	*Cm*LeuRS CP1 + Nva2AA	*Cm*LeuRS CP1 + Ile2AA	*Cm*LeuRS CP1 + AN6426-AMP
Data collection statistics				
Beam line	Soleil, France	ESRF, France (ID23-1)	SSRF, China	Soleil, France
Space group	P4_1_2_1_2	P4_1_2_1_2	P4_3_2_1_2	P4_3_2_1_2
Cell dimensions				
*a*, *b*, *c* (Å)	107.71, 107.71, 311.20	107.73, 107.73, 309.51	64.93, 64.93, 167.25	65.07, 65.07, 167.30
α, β, γ (°)	90.00, 90.00, 90.00	90.00, 90.00, 90.00	90.00, 90.00, 90.00	90.00, 90.00, 90.00
Resolution (Å)	48–2.70 (2.70–2.84)	48–2.30 (2.30–2.36)	50–1.76 (1.76–1.79)	60–2.10 (2.10–2.21)
*R*_sym_	4.8 (93.4)	11.3 (129.0)	17.2 (121.0)	4.4 (47.3)
*I*/σ*I*	30.3 (2.7)	16.5 (1.9)	19.5 (2.1)	29.2 (5.6)
Completeness (%)	99.8 (99.0)	99.9 (99.3)	99.7 (95.3)	99.9 (99.4)
Redundancy	8.7 (9.0)	8.1 (7.9)	23.8 (18.6)	10.4 (10.6)
Refinement statistics				
Resolution (Å)	2.7	2.3	1.7	2.10
No. of reflections work/free	48,772/2,609	77,686/4,014	29,524/1,573	20,768/1,122
*R*_work_/*R*_free_	0.209/0.253	0.201/0.236	0.196/0.232	0.200/0.238
No. of:				
Protein atoms	8,683^*c*^	8,892^*c*^	2,235	2,185
Ligands		104^*d*^	27^*e*^	38^*f*^
Ions		2 (K^+^)	10 (2× PO_4_^2−^)	5 (PO_4_^2−^)
Water molecules/other	71	371/16^*g*^	232	51
*B* factor				
Protein	90.1	47.2	24.3	54.9
Ligand		64.3	17.3	50.5
Ions		43.4 (K^+^)	46.6 (PO_4_^2−^)	98.9 (PO_4_^2−^)
Water/other	65.7	42.8/50.8 (EG^*h*^)	34.8	51.6
RMSD				
Bond lengths (Å)	0.010	0.009	0.014	0.009
Bond angles (°)	1.41	1.38	1.67	1.61

a $R_{\text{sym}}=\frac{\Sigma_{hkl}\Sigma_{j}\left|I_{hkl,j}-〈I_{hkl}〉\right|}{\Sigma_{hkl}\Sigma_{j}I_{hkl,j}}$, where $〈I_{hkl}〉$ is the average of symmetry (or Friedel)-related observations of a unique reflection; *I*, intensity of a reflection.

b Values in parentheses are for the highest-resolution shell.

c Four subunits.

d Four Nva2AA molecules.

e Ile2AA.

f AN6426-AMP.

g Four ethylene glycol molecules.

h EG, ethylene glycol.

**FIG 2:**
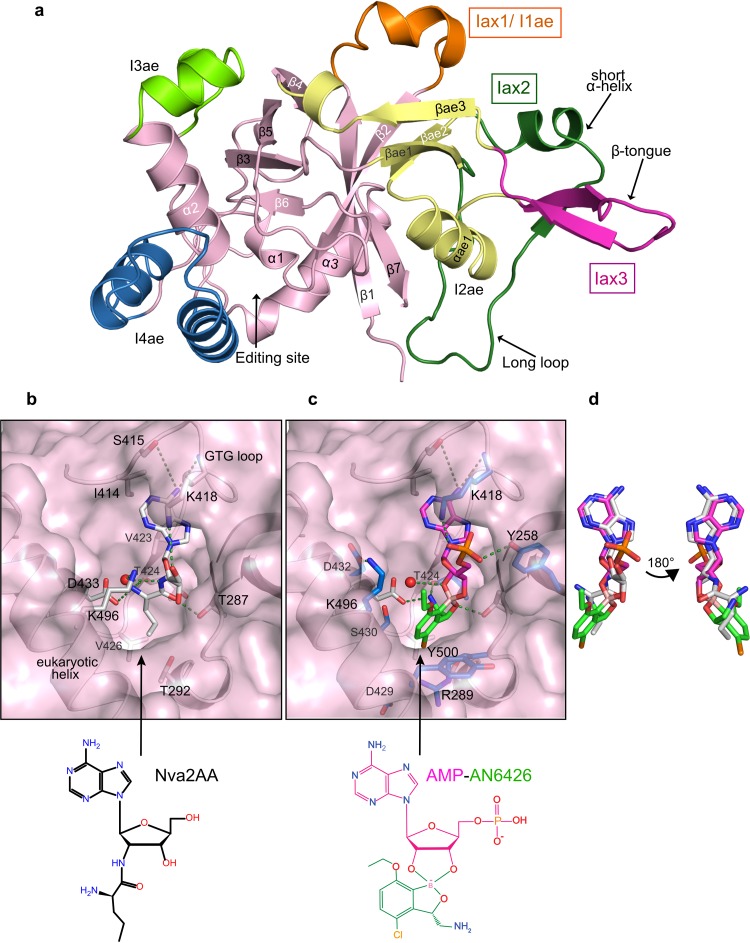
Crystal structures of posttransfer editing analogues and AN6426 with *Cm*LeuRS. (a) Crystal structure of apo-*Cm*LeuRS showing the canonical editing domain core in pink and the eukaryotic insertions in orange for I1ae, yellow for I2ae, and chartreuse for I3ae. Apicomplexa-specific insertions are highlighted with labels in boxes. Secondary structural elements contained in the apicomplexa insertions are indicated by arrows. (b) Posttransfer editing analogue of norvaline (Nva2AA) bound into the editing site of *Cm*LeuRS. The analogue and key protein residues that are important for editing or binding of Ade76 are shown as white sticks and labeled. Key hydrogen bonds between the protein and Nva2AA are depicted by green dashed lines, and the water molecule involved in hydrolysis is shown as a red sphere. (c) The AN6426-Ade76 (AMP) adduct (shown as pink sticks for AMP and green sticks for AN6426) bound into the editing site of *Cm*LeuRS. Key residues that contact the adduct are shown as sticks, but only residues showing additional interactions with respect to editing analogues are colored in blue and labeled. (d) The conformation of the AN6426-Ade76 adduct mimics the conformation of the posttransfer editing analogue of norvaline bound into the editing site of *Cm*LeuRS. For clarity, the overlap is shown both in the same orientation as in panels b and c and in an orientation rotated 180 degrees.

The structures show the typical seven-β-strand and three-α-helix fold of the LeuRS connective polypeptide domain (CP1) (41, 49), plus archaeon/eukaryote-specific insertions, denoted I1ae, I2ae, I3ae, and I4ae (43). In addition, *Cm*LeuRS has three apicomplexa-specific insertions, which we denote Iax1, Iax2, and Iax3 (Fig. 2a; see Fig. S2 in the supplemental material for the sequence alignment). Despite predicted flexibility, the three insertions are, in fact, ordered and include well-defined secondary structure elements. Iax1 of *Cm*LeuRS is rather short compared to the length of homologous insertions in other apicomplexan species, such as Plasmodium, and partially overlaps a helix of I1ae (Fig. 2a). Iax2 protrudes from β-sheets βae1 and βae2 of I2ae and contains an 8-residue α-helix followed by a short β-strand and a long loop. Iax3 consists of a β-tongue structure which connects the α-helix (αae1) and the third β-sheet (βae3) of I2ae. Interestingly, Iax3 has a well-defined β-sheet in the apo-structure, whereas all the holo-structures show a significantly different conformation with shifts of up to 8.9 Å (see Fig. S3 in the supplemental material). Another difference between the apo- and holo-structures is the position of helices α1 and α2 adjacent to I4ae at the editing site, which needs to open (∼1.5 Å) to accommodate ligands. Overall, the *Cm*LeuRS insertions are 25 to 45 Å distant from the editing site (see Fig. S3a in the supplemental material) and thus are presumably not directly involved in posttransfer editing, although we cannot exclude this possibility in the context of the full-length protein and tRNA. Whether the insertions mediate noncanonical AARS functions, as has been previously found for other insertions (reviewed in references 50 and 51), remains to be investigated.

The posttransfer editing substrate analogues Nva2AA and Ile2AA can be unambiguously placed at the editing site of *Cm*LeuRS (see Fig. S4a and b in the supplemental material). As was observed for bacterial and fungal LeuRSs (43, 52), the aliphatic part of the amino acid binds into the hydrophobic pocket formed by residues T292 and V426, while the amino group interacts with the universally conserved aspartic acid (D433); the adenine base is accommodated by residues of the GTG loop (I414, S415, V423, and K418) (Fig. 2b). In our structures, a water molecule is well positioned for promoting hydrolysis (3 to 4 Å to the carbon alpha atom) and is coordinated by a eukaryote-specific lysine (K496) (Fig. 2b; see also Fig. S2 in the supplemental material). A similarly placed water molecule is important for bacterial LeuRS editing complexes, although this is coordinated by a bacterium-specific aspartic acid (D344 in the Thermus thermophilus LeuRS). This suggests a common editing mechanism via nucleophilic attack of this water on the oxygen carbonyl (see Fig. S5a and b in the supplemental material), consistent with quantum mechanical calculations on LeuRS (53). However, the mechanism could be more complex than was previously thought, as it is unclear how an aspartate or a lysine with different pK_a_ values could play an equivalent role in hydrolysis. Interestingly, a lysine residue in the editing domain of class II ThrRS plays a key role in hydrolysis by coordinating a catalytic water molecule, which is equivalent to the water molecule that we observed in *Cm*LeuRS (see Fig. S5c in the supplemental material). Such a lysine (K121 in Pyrococcus abyssi ThrRS) excludes the catalytic water molecule in a complex with the cognate Thr analogue, thereby discriminating cognate threonine-tRNA at the editing site (54, 55). The interactions of the catalytic water and K496 at the editing site of *Cm*LeuRS are strikingly similar to the situation in the ThrRS-Ser3AA complex (see Fig. S5a and c in the supplemental material), which suggests that K496 could have a similar role in hydrolysis by eukaryotic LeuRS.

### Structure of inhibition complex of *Cm*LeuRS bound to AN6426-AMP.

**FIG 3:**
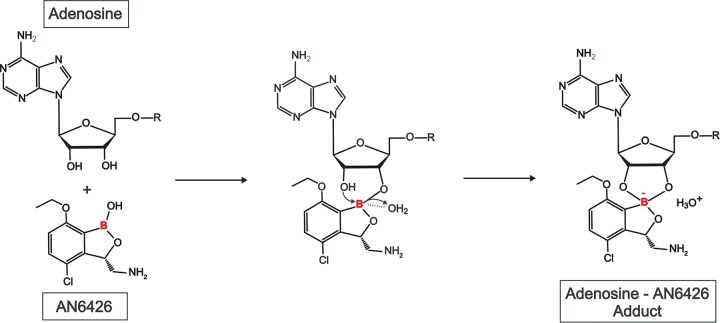
Formation of AN6426-adenosine adduct. Proposed mechanism of formation of the adenosine adduct with the inhibitor AN6426. The reaction proceeds via a first step in which the 2′- or 3′-hydroxyl of the adenosine ribose reacts with the boron atom, followed by a second step in which the remaining hydroxyl, most probably stabilized by a water molecule (observed in the crystal structure), reacts with the boron atom, which adopts an sp3 tetrahedral hybridization, and forms the final pentyl cycle observed in the crystal structure.

The structure of the *Cm*LeuRS-AN6426-AMP complex shows that the inhibitor and AMP (Ade76 mimic) form a covalent adduct that binds tightly in the editing active site, occupying the same site as posttransfer editing substrates (Fig. 2c and d; see also Fig. S3b to d in the supplemental material). We hypothesize that adduct formation occurs by an initial reaction of either the 2′- or 3′-hydroxyl of tRNA with the boron atom of AN6426, a state probably stabilized by a water molecule, followed by nucleophilic attack of the remaining hydroxyl, leading to the formation of the 5-membered-ring adduct observed in the crystal structure (Fig. 3). The adenosine occupies a position nearly identical to that in the complexes with Nva2AA or Ile2AA. However, AN6426 establishes additional contacts, hence explaining the higher affinity of AN6426 for the enzyme (Fig. 2b and c). The 3-aminomethyl group of AN6426 mimics the amino group of norvaline and establishes hydrogen bonds to T424 and D433, the Cl 4 of AN6426 mimics the delta carbon of norvaline/isoleucine and makes van der Waals contacts with V426 and T292, and the oxygen 1 of AN6426 mimics the hydrogen bond of T287 to the carbonyl oxygen of norvaline/isoleucine (Fig. 2b and c). The additional interactions of AN6426 are hydrophobic contacts by carbons 4 to 6 with the aliphatic atoms of R289, D429, and S430, and a hydrogen bond between the phosphate oxygen of Ade76 and Y258 (Fig. 2b and c). Compared to the structure of the *Cm*LeuRS-AN6426-AMP complex, the posttransfer analogues retain 3 to 4 extra water molecules (depending on the monomer chain) and a phosphate (*Cm*LeuRS-Ile2AA complex), which mediate polar interactions between the compounds and protein residues. These solvent molecules are released upon binding of AN6426, most likely due to hydrophobic exclusion by the ethoxy group and carbons 4 to 6 of AN6426, and likely contribute to affinity with a favorable desolvation entropy. This, together with the absence of conformational changes upon ligand binding (RMSD of the apo-structure versus the holo-structure of AN6426-AMP, 0.59 Å), explains the favorable entropy and the significant difference from bacterial LeuRS inhibitors, which are characterized not only by hydrophobic interactions but also by additional polar contacts (Fig. 1).

Interestingly, our structures show that, compared to bacterial LeuRS, the eukaryote-specific helix (residues 489 to 507) considerably restricts the available volume of the editing site, and this knowledge can help guide the design of future *Cm*LeuRS inhibitors. Specifically, residues K496 and Y500 need to adjust their conformation to accommodate the ethoxy group of AN6426. Of note, the side chain of K496 is shifted 4 Å compared to its location in the complexes with Nva2AA and Ile2AA and is stabilized in its new orientation by forming a salt bridge with D432 (Fig. 2b and c). Therefore, the introduction of big aliphatic groups into positions 5 and 6 of AN6426 most likely leads to clashes with these residues, explaining the lack of binding that we previously observed for such derivatives (data not shown).

### AN6426 has efficacy against Toxoplasma gondii in human fibroblasts.

**FIG 4:**
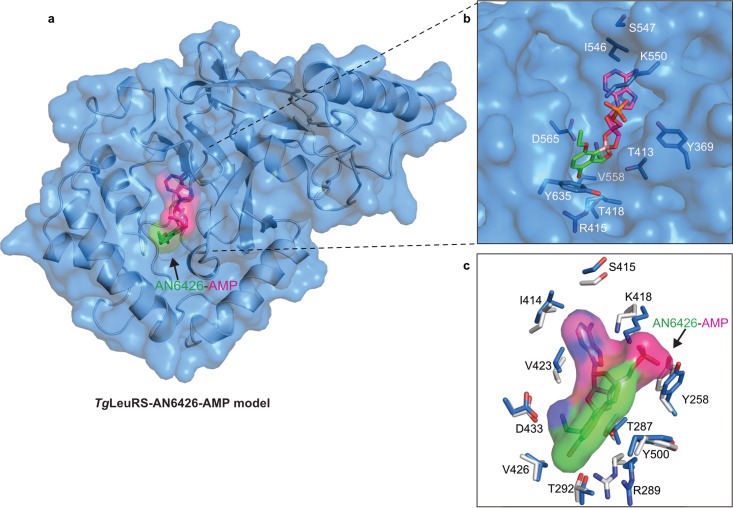
*Tg*LeuRS-AN6426-AMP model and conservation of residues. (a) Homology model of *Tg*LeuRS calculated by structural alignment and subsequent model building using as a template the editing domain of the *Cm*LeuRS complex with AN6426-AMP determined in this work (see Materials and Methods). The protein model is shown in a carton-and-surface representation (blue); the inhibition adduct is shown in a sticks-and-surface representation, with the AMP moiety being shown in pink and AN6426 being shown in green. (b) Magnification of the view of AN6426-AMP in the editing site of *Tg*LeuRS, which shows no steric clashes with residues that are key to the interaction with AN6426-AMP (shown as blue sticks). (c) Conservation of residues between the editing sites of *Cm*LeuRS and *Tg*LeuRS. Key residues in the interaction with AN6426-AMP (shown in a sticks-and-surface representation) are shown as blue sticks for *Tg*LeuRS and white sticks for *Tg*LeuRS. For clarity, only residues of *Cm*LeuRS are labeled. The figure is rotated 180 degrees with respect to the orientation in panel b.

Using sequence and structural alignments, we built a homology model of the T. gondii LeuRS editing domain based on that of Cryptosporidium LeuRS (Fig. 4a; see also Fig. S2 in the supplemental material) and docked the AN6426-AMP adduct into its editing site by superposition with the structure of *Cm*LeuRS-AN6426-AMP (RMSD for backbone atoms, 1.27 Å) (Fig. 4b). No structural constraints or steric clashes with the AN6426-AMP adduct were observed, consistent with the high degree of conservation of residues at the editing site (Fig. 4c). We thus hypothesized that the Toxoplasma LeuRS could also be a suitable target for inhibition by AN6426 or related compounds.

**FIG 5:**
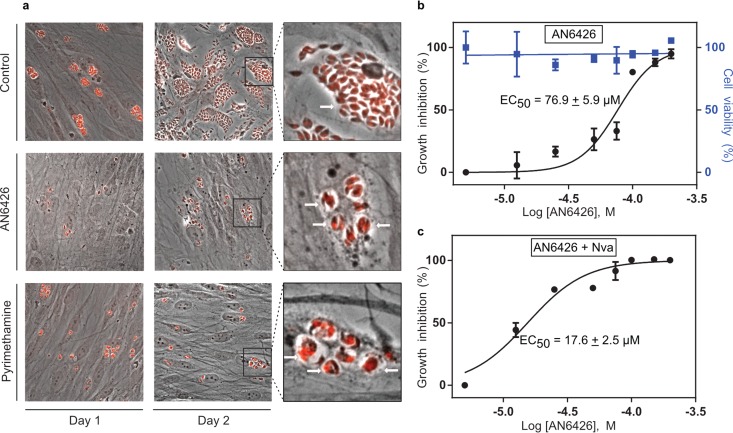
Efficacy of AN6426 against T. gondii in human cells and norvaline effect. (a) Immunofluorescence microscopy experiments monitoring the proliferation of parasites upon infection of human fibroblasts. Parasites were stained with specific antibodies directed against sumoylated parasite proteins (red). Comparison of untreated cells (negative control) at 24 and 48 h postinfection with cells treated with AN6426 (100 μM) or pyrimethamine (2 μM) shows a reduction of the parasite load in human cells. The insets on the right show higher-magnification views of parasitic vacuoles, with untreated cells containing >40 parasites per vacuole and AN6426-treated cells containing 2 to 4 parasites per vacuole (i.e., after 1 or 2 replication cycles). Single representative vacuoles are indicated by arrows. Treatment with AN6426 was carried out for 4 days starting at 8 h postinfection (see Fig. S6 in the supplemental material for more details). (b and c) Dose-response curves showing the activities of AN6426 (b) and AN6426 in the presence of norvaline (Nva) (c) against T. gondii and HFF viability. The dose-response curves were normalized by the ratio of parasites/vacuoles for the untreated cells. EC_50_s were calculated by fitting the data to a sigmoidal dose-response inhibition model. The data are the means from three independent measurement, and the error bars indicate the standard deviations.

To test our hypothesis, we monitored parasite growth in human foreskin fibroblasts (HFFs) hosting T. gondii tachyzoites and initiated treatment with AN6426 at 8 h postinvasion. At 2 days postinfection, untreated cells were lysed due to the massive proliferation of the parasites (Fig. 5a; see also Fig. S6 in the supplemental material). In contrast, cells treated with AN6426 exhibited a significant reduction in parasite burden, with an EC_50_ of 76.9 μM (Fig. 5a and b). Interestingly, AN6426-treated parasites replicated only once or twice (2 to 4 parasites per vacuole); in contrast, 40 to 60 parasites per vacuole were found in the control (Fig. 5a). No apparent toxicity of AN6426 to primary human cells was observed at concentrations up to 200 μM.

To further evaluate the target of AN6426 in T. gondii, we compared the activity of AN6426 in the presence and absence of norvaline, a nonstandard amino acid that, when mischarged to the 3′ end of tRNA^Leu^, is a substrate for posttransfer editing by LeuRS. Recent experiments have shown that LeuRS posttransfer editing activity is critical for Escherichia coli survival under certain stress conditions, such as oxygen starvation, which leads to increased norvaline concentrations in E. coli (45, 56). Contrary to the findings for Cryptosporidium, in which only limited development occurs in cell cultures, Toxoplasma completes multiple replication cycles (every 6 to 8 h), enabling investigation of the long-term effects of norvaline. If AN6426 binds to and inactivates the editing site of *Tg*LeuRS, for example, by forming a tightly binding adduct with ATP, one would expect norvaline to have detrimental effects on parasite survival. Norvaline alone (up to 10 mM) was not toxic to T. gondii or human cells, suggesting a robust LeuRS editing activity. However, when combined with 10 mM norvaline, AN6426 had a 5-fold decrease in its EC_50_ compared to the value obtained in the absence of norvaline (Fig. 5b and c). These results are consistent with AN6426 targeting the editing site of *Tg*LeuRS but do not rule out the possibility that AN6426 targets other Toxoplasma proteins.

## DISCUSSION

In this work, we show that the benzoxaborole AN6426 inhibits growth in human cells of two apicomplexan parasites, C. parvum (very close in sequence to C. hominis, the main human pathogen) and T. gondii, and we provide evidence that the target of AN6426 in both organisms is the LeuRS editing site. AN6426 was active against C. parvum in MDCK cells, with an EC_50_ of 2.2 μM, which represents a more than 230-fold improvement compared to that of paromomycin, a drug active against Cryptosporidium (3). *In vitro* binding experiments and cocrystal structures with *Cm*LeuRS showed that the AN6426-AMP adduct binds to the editing site with a higher affinity than the posttransfer editing substrates (Fig. 1 to 2). In the case of T. gondii, AN6426 prevented proliferation of parasites in human fibroblasts at midmicromolar concentrations. However, a 5-fold decrease in the EC_50_ was observed in the presence of 10 mM norvaline, consistent with the inhibitor blocking the LeuRS editing site and thus perturbing the editing activity of LeuRS. Furthermore, these studies provide evidence that posttransfer editing occurs in T. gondii and open the way to the design of more potent inhibitors of the *Tg*LeuRS editing site.

The findings presented above are consistent with the inhibition mechanism demonstrated previously for other benzoxaboroles with efficacy against fungi (16) and bacteria (15, 57) and also likely against Plasmodium falciparum (23). This involves the formation of a stable covalent adduct (Fig. 3) of AN6426 in the LeuRS editing site that can block aminoacylation if it interacts with the tRNA^Leu^ acceptor end or block posttransfer editing if it interacts with, for instance, ATP.

Our thermodynamic analysis revealed that the mode of AN6426 binding to *Cm*LeuRS differs from that to bacterial LeuRS. Polar contacts are less important for the interaction with *Cm*LeuRS, consistent with the additional hydrophobic contacts observed in the structure of its complex with AN6426-AMP. In addition, our structures indicate that the editing site of *Cm*LeuRS has a binding cavity smaller than that of prokaryotic LeuRSs. These findings will facilitate the design of more potent and selective inhibitors. In this context, the good correlation between AN6426 *in vitro* and *ex vivo* parameters (*K_d_* = 9.8 μM versus EC_50_ = 2.2 μM) will facilitate the rapid characterization of new compounds.

In recent years, AARSs have become the focus of several studies to identify new inhibitors with therapeutic applications. Some compounds have shown good antiparasitic activity *in vitro* and efficacy in animal models (reviewed in reference 18). Of note were (i) halofuginone, an herbal extract that targeted the ProRS active site (58–60) but that, unfortunately, was hepatotoxic (61); (ii) cladosporin and lysyl-adenylate analogues as inhibitors of P. falciparum LysRS, which were not orally bioavailable (62, 63); and (iii) mupirocin and isoleucine analogues, which inhibited *in vitro* P. falciparum IleRS (64, 65). The demonstration that AN6426 is active against Cryptosporidium, Toxoplasma, and P. falciparum (23) suggests that LeuRS is a valid target in these parasites. This conclusion, combined with the good pharmacokinetic and pharmacodynamic properties of these compounds, including good oral bioavailability (15) and apparent low toxicity (66), suggests that benzoxaboroles targeting apicomplexan parasites merit further development.

## Supplementary Material

Supplemental material
