# Supplementary material for: Cryptosporidium and Toxoplasma Parasites Are Inhibited by a Benzoxaborole Targeting Leucyl-tRNA Synthetase
Source: Antimicrob Agents Chemother. 2016 Sep 23;60(10):5817–27. doi: 10.1128/AAC.00873-16 (PMC5038320; doi:10.1128/AAC.00873-16)
Supplement: Supplemental material [file supp_60_10_5817__index.html]

Cryptosporidium and Toxoplasma Parasites Are Inhibited by a Benzoxaborole Targeting Leucyl-tRNA Synthetase — Supplemental material 

# Cryptosporidium and Toxoplasma Parasites Are Inhibited by a Benzoxaborole Targeting Leucyl-tRNA Synthetase

## Supplemental material

- Supplemental file 1 -

  Supplemental Table S1 and Figures S1 to S6.

  PDF, 5.3M
