# Supplementary material for: Cryptosporidium and Toxoplasma Parasites Are Inhibited by a Benzoxaborole Targeting Leucyl-tRNA Synthetase
Source: Antimicrob Agents Chemother. 2016 Sep 23;60(10):5817–27. doi: 10.1128/AAC.00873-16 (PMC5038320; doi:10.1128/AAC.00873-16)
Supplement: Supplemental material [file AAC.00873-16_zac010165527so1.pdf]

SUPPLEMENTAL MATERIAL

Cryptosporidium and *Toxoplasma* parasites are inhibited by a benzoxaborole targeting leucyl-tRNA synthetase

Andrés Palencia, Ru-Juan Liu, Maria Lukarska, Jiri Gut, Alexandre Bougdour, Bastien Touquet, En-Duo Wang, Xianfeng Li ,M.R.K. Alley, Yvonne R. Freund, Philip J. Rosenthal, Mohamed-Ali Hakimi and Stephen Cusack

Table S1. Binding energetics of compounds to *C. muris* LeuRS

Name	Structure	*K_d (μM)	ΔG_{app} (Kcal mol^{-1})	ΔH_{app} (Kcal mol^{-1})	$-T\Delta S_{app}$ (Kcal mol^{-1})
Nva2AA	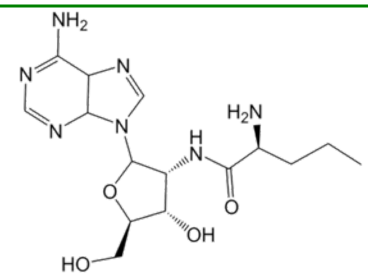	213±4	-5.0	-1.7	-3.3
Ile2AA	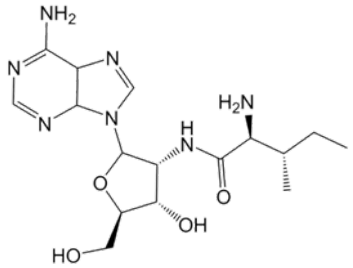	215±7	-5.0	-0.3	-4.7
AN6426	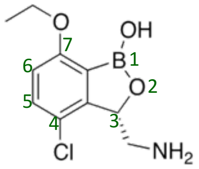	9.8±0.3	-6.8	5.0	-11.8

* Values represent the mean from at least two independent experiments and errors are the standard deviations. The errors in the binding enthalpies are about 10%.

Supplementary Figure 1. Determination of EC_{50} values against *C. parvum*. Dose-response curves showing the activity of AN6426 **(a)** and AN8432 **(b)** against *C. parvum* in MDCK cells. The dose-response curves were normalized to 0 % (bottom) and 100 % (top) inhibition growth. Hill slope was constrained to be < 5. EC_{50} values were calculated by fitting the data of two independent experiments, top and bottom panels in (a) and (b). Calculated EC_{50} values and standard deviations are shown in **Table 1**.

a **b**

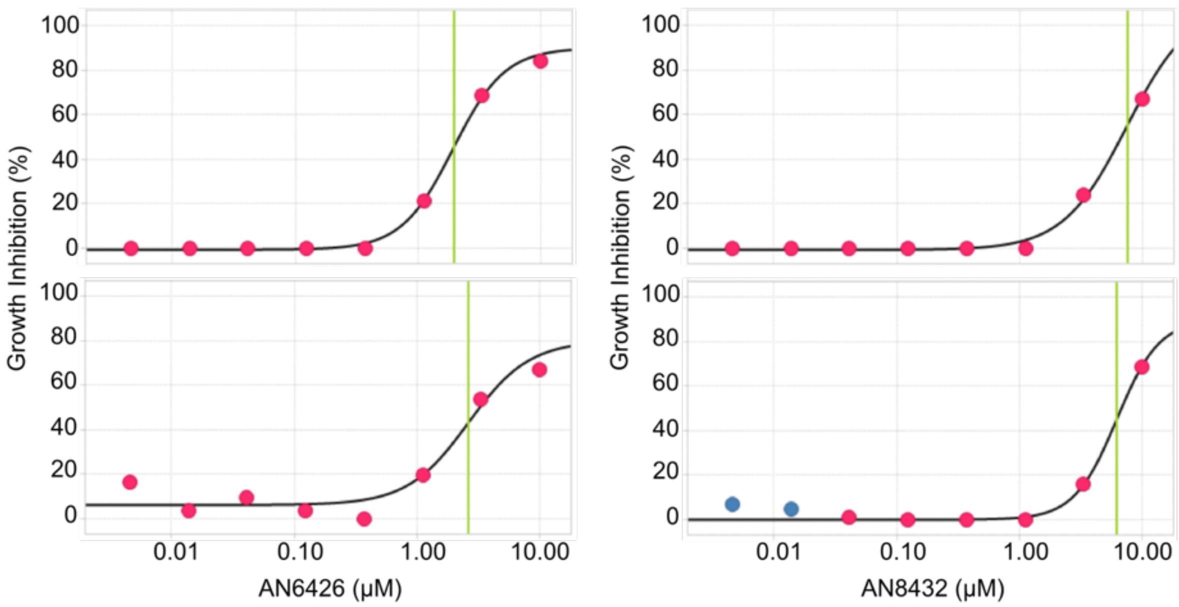

Supplementary Figure 2. Sequence alignment of eukaryotic LeuRSs. Sequence alignment of representative eukaryotic LeuRSs with partial conservation outlined in same color. The predicted N-terminal and C-terminal regions of the editing domain of *Cryptosporidium muris* LeuRS are indicated with arrows. The 3 insertions that are specific to apicomplexan parasites are denoted by Iax1, Iax2, and Iax3, and are underlined with colored boxes. Key residues involved in hydrolysis of post-transfer editing substrates or in discrimination of cognate leucine are marked with stars.

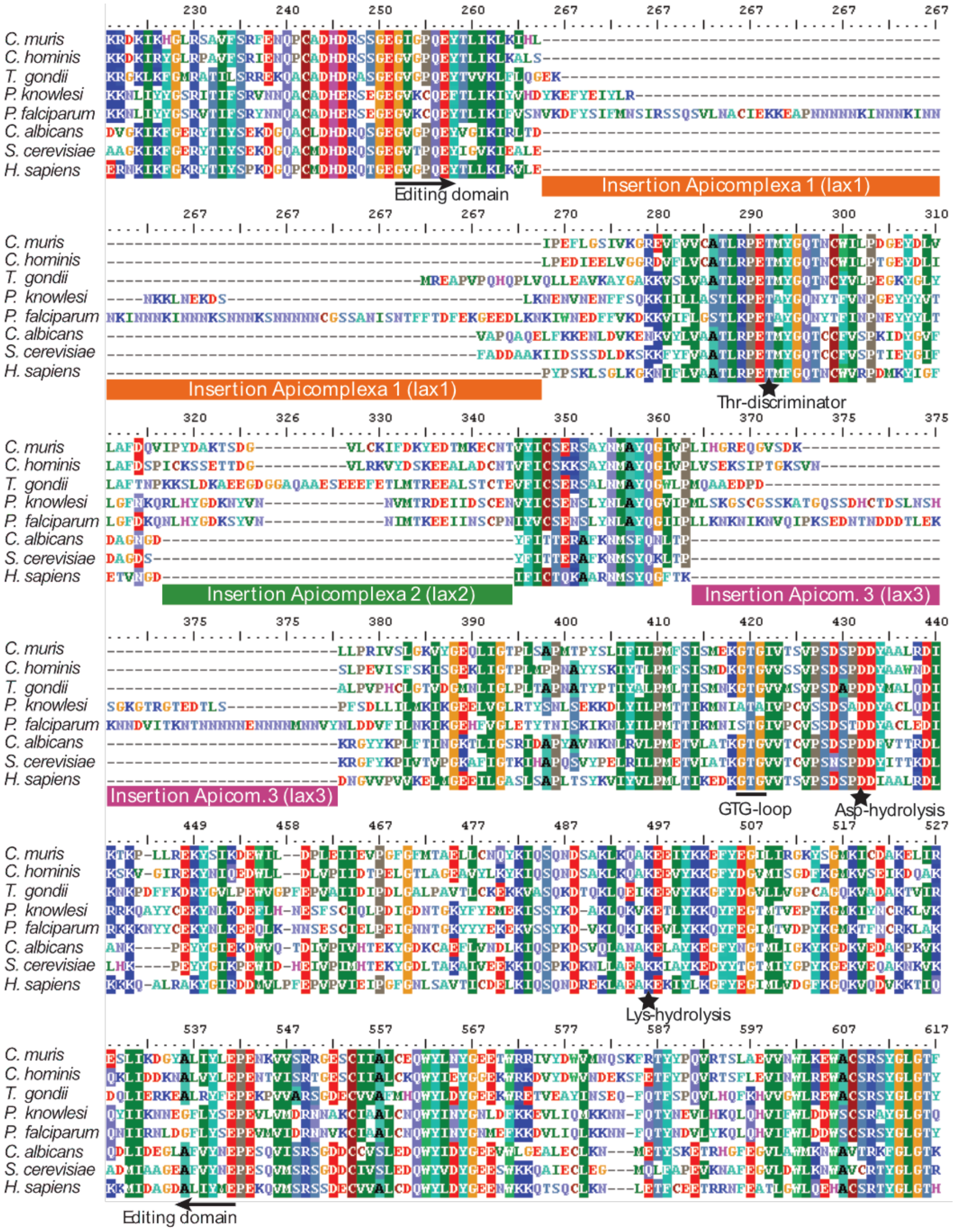

Supplementary Figure 3. Summary of *Cm*LeuRS X-ray structures. Crystal structures of the **(a)** editing domain of cytosolic *Cm*LeuRS (unbound), **(b)** *Cm*LeuRS complex with post-transfer editing analogue of norvaline (Nva2AA), **(c)** *Cm*LeuRS complex with post-transfer editing analogue of isoleucine (Ile2AA), and **(d)** *Cm*LeuRS complex with the adduct AN6426–Ade76 bound into the editing active site. The color code is as shown in **Fig 2a**. Arrows in panel **(a)** indicate differences between the apo structure of *Cm*LeuRS editing domain and the holo-structures. A discontinuous arrowed-line shows a possible correlated motion between the editing site and insertion Iax3. Insets show a zoomed view of the different ligands into the editing site of *Cm*LeuRS.

a

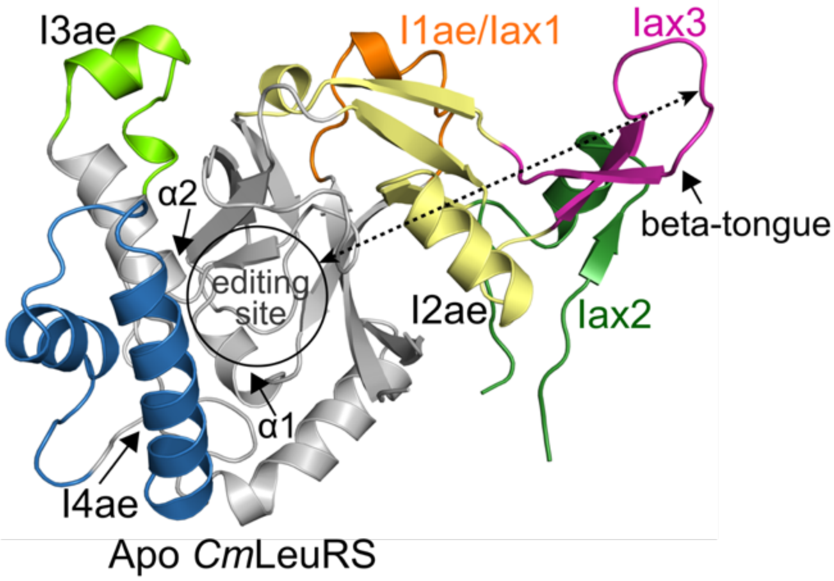

b

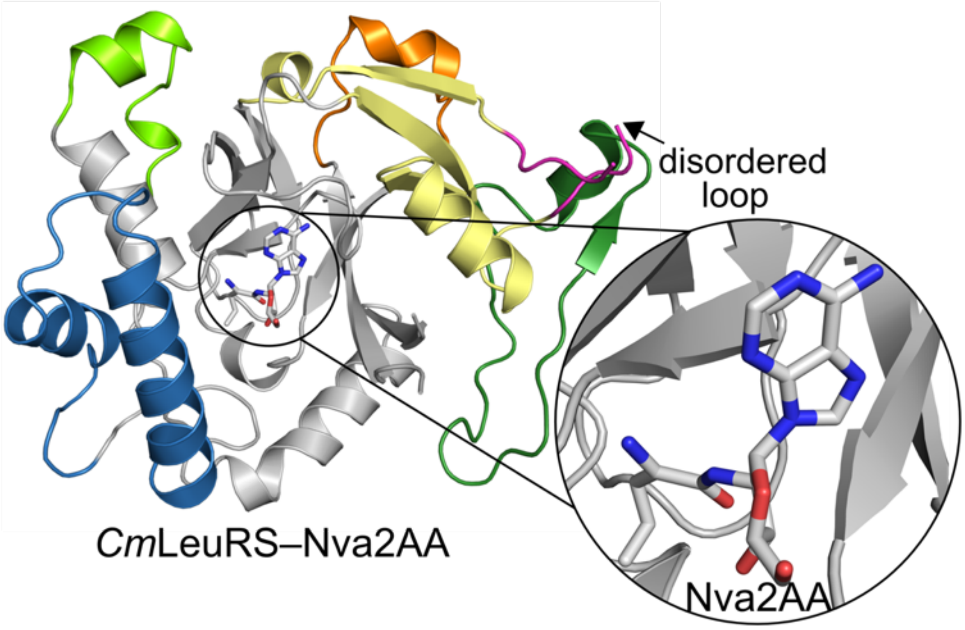

c

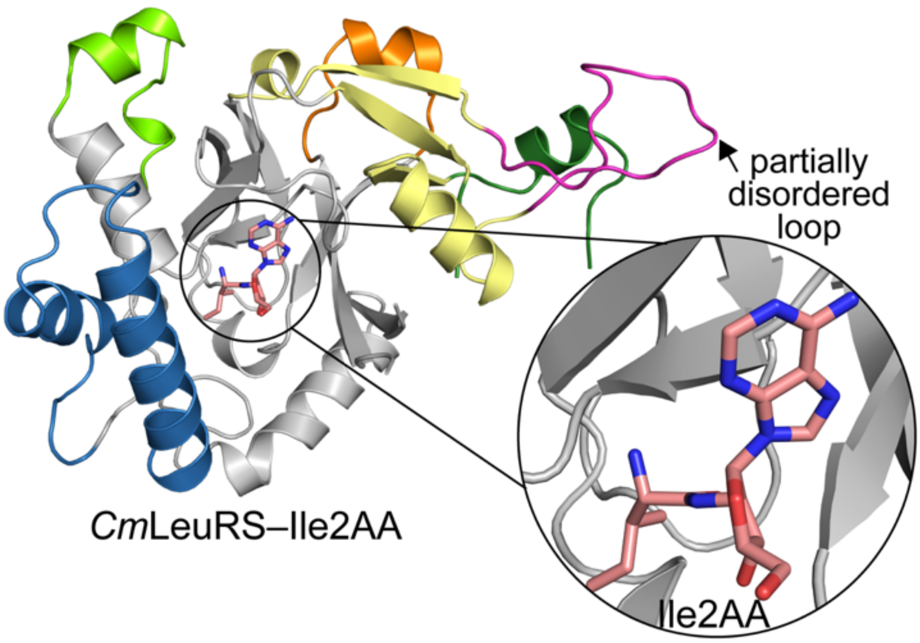
partially
disordered
loop
*Cm*LeuRS–Ile2AA
Ile2AA

d

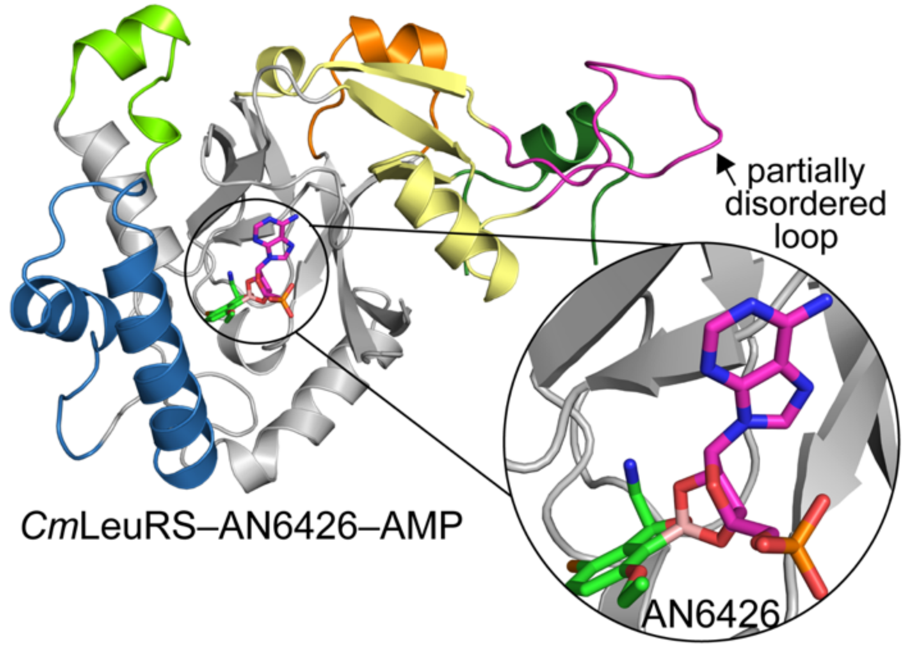
partially
disordered
loop
*Cm*LeuRS–AN6426–AMP
AN6426

Supplementary Figure 4. Unbiased electron density difference maps. Stereo diagrams showing unbiased m(Fo-Fc) difference electron density (contoured at 3.5 σ) for (**a**) post-transfer editing substrate analogue of norvaline (Nva2AA) bound into the editing site of *Cm*LeuRS; **(b)** post-transfer editing substrate analogue of isoleucine (Ile2AA) bound into the editing site of *Cm*LeuRS; and **(c)** the adduct formed by the benzoxaborole AN6426 with adenosine monophosphate (mimic of Ade76 tRNA base) at the editing site of *Cm*LeuRS. The density was obtained by rebuilding and refinement of the molecular replacement model without inclusion of the relevant analogue or adduct.

a

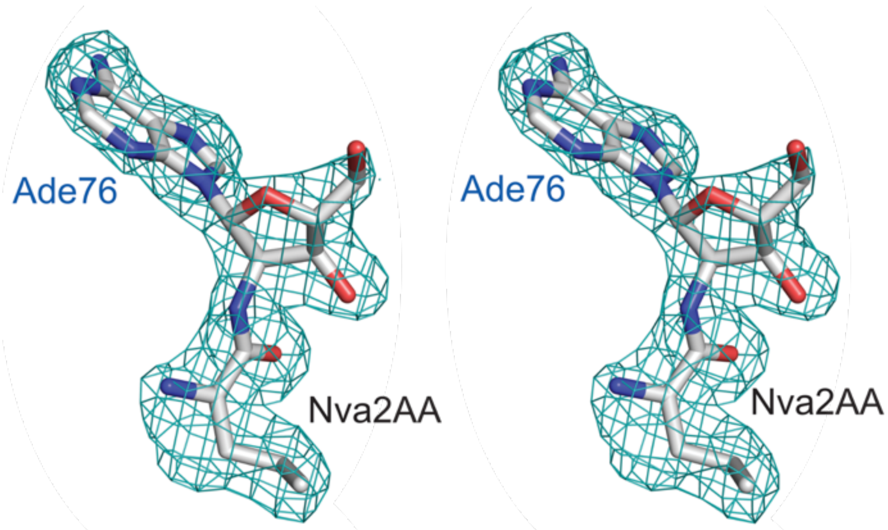
Ade76
Nva2AA
Ade76
Nva2AA

b

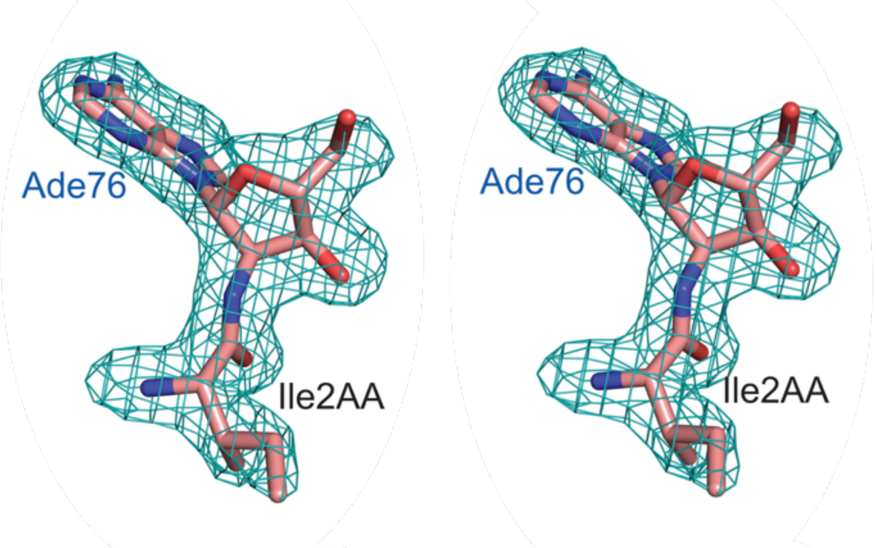
Ade76
Ile2AA
Ade76
Ile2AA

c

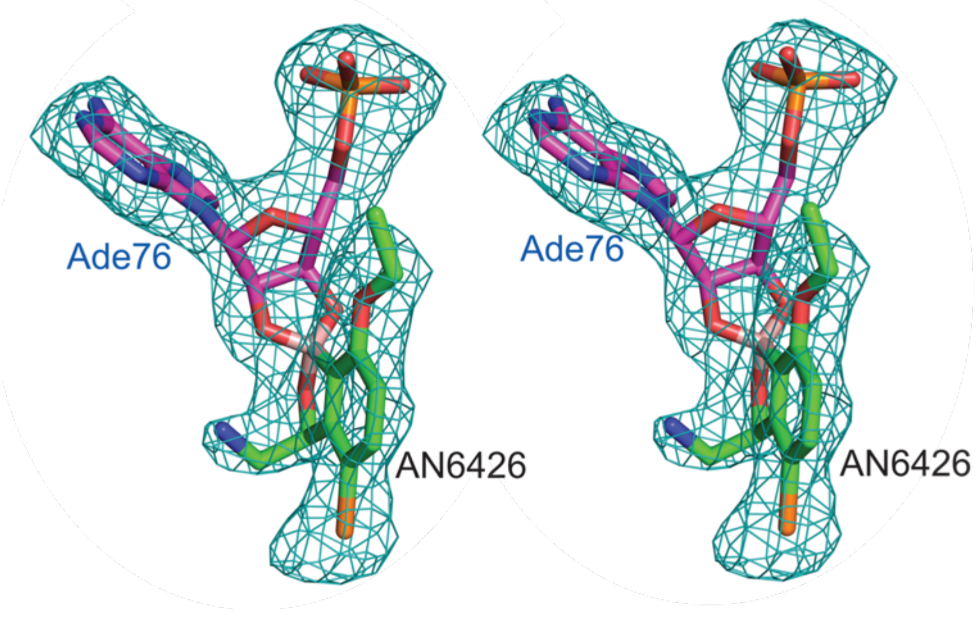
Ade76
AN6426
Ade76
AN6426

Supplementary Figure 5. Catalytic water molecule at the editing site of LeuRSs and ThrRS.

(a) Post-transfer editing analogue of norvaline (Nva2AA) bound into the editing site of *Cm*LeuRS and the eukaryotic specific lysine (496) are shown as white sticks. **(b)** Post-transfer editing analogue bound into the editing site of *T.thermophilus* LeuRS (PDB:1OBC) and bacterial specific aspartic acid (D344 in *T.thermophilus*) are shown as pink sticks. **(c)** Post-transfer editing analogue of threonine (Ser3AA) bound into the editing site *P.abysii* ThrRS (PDB:3PD2) and the highly conserved lysine residue (K121 in *P.abysii*) are shown as green sticks. K121 coordinates the catalytic water molecule involved in hydrolysis of post-transfer editing substrate of serine. This catalytic water molecule (shown as red sphere) is observed at equivalent positions in eukaryotic/bacterial LeuRS, panel **(a)** and **(b)** respectively. Calculated electrostatic potential surfaces are shown in top panels, and key interactions between protein residues and the editing substrates that are mediated by the catalytic water molecule are shown as green-dashed lines.

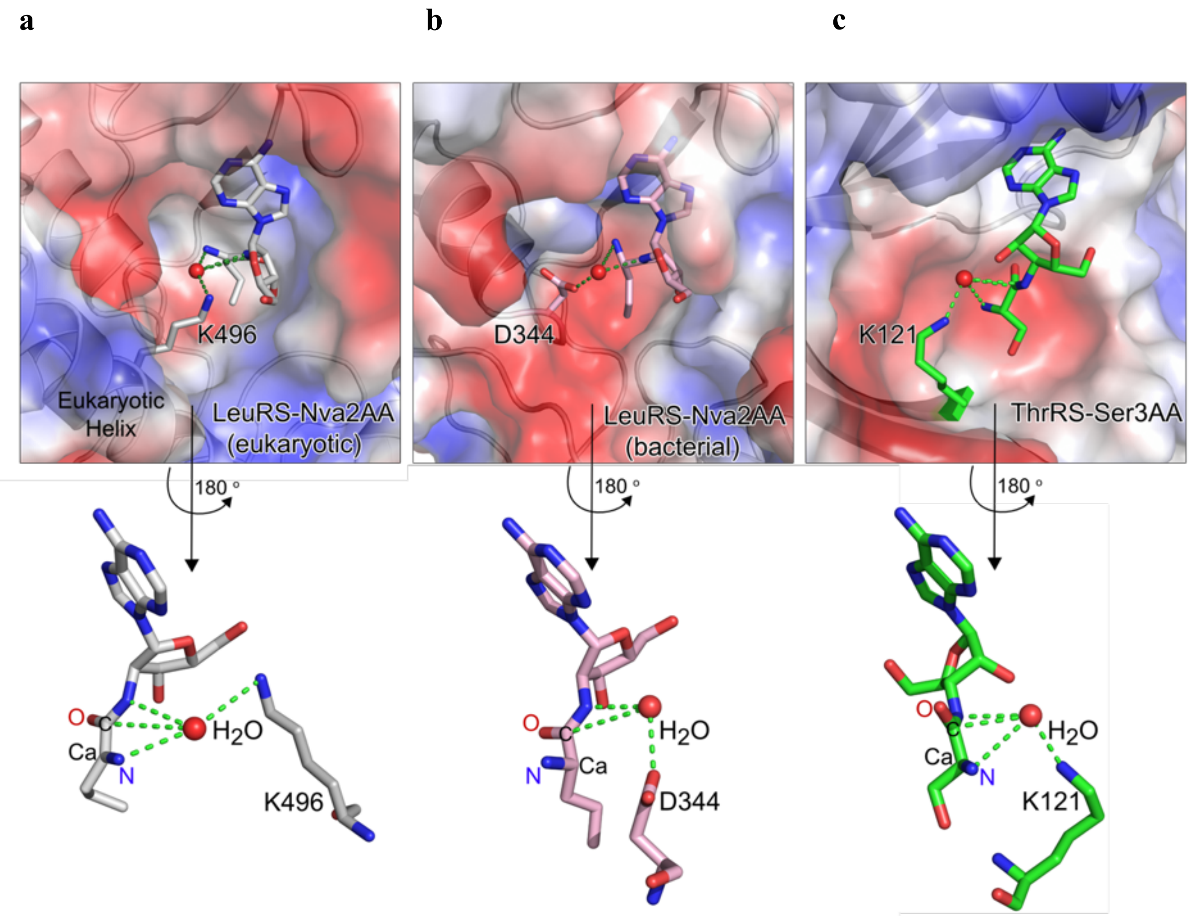
a
b
c
K496
Eukaryotic Helix
LeuRS-Nva2AA (eukaryotic)
D344
LeuRS-Nva2AA (bacterial)
K121
ThrRS-Ser3AA
180 °
180 °
180 °
O
C
Ca
N
H2O
K496
O
C
N
Ca
H2O
D344
O
C
Ca
N
H2O
K121

Supplementary Figure 6. Immunofluorescence assays showing the efficacy of AN6426 against *T. gondii* parasites in human fibroblasts. Infection of un-treated cells (control) leads to lysis of fibroblasts due to uncontrolled proliferation of parasites at 48-hours post-infection. AN6426 has efficacy to inhibit parasite growth. Treatment of infected human cells with AN6426 started 8 hours post-infection.

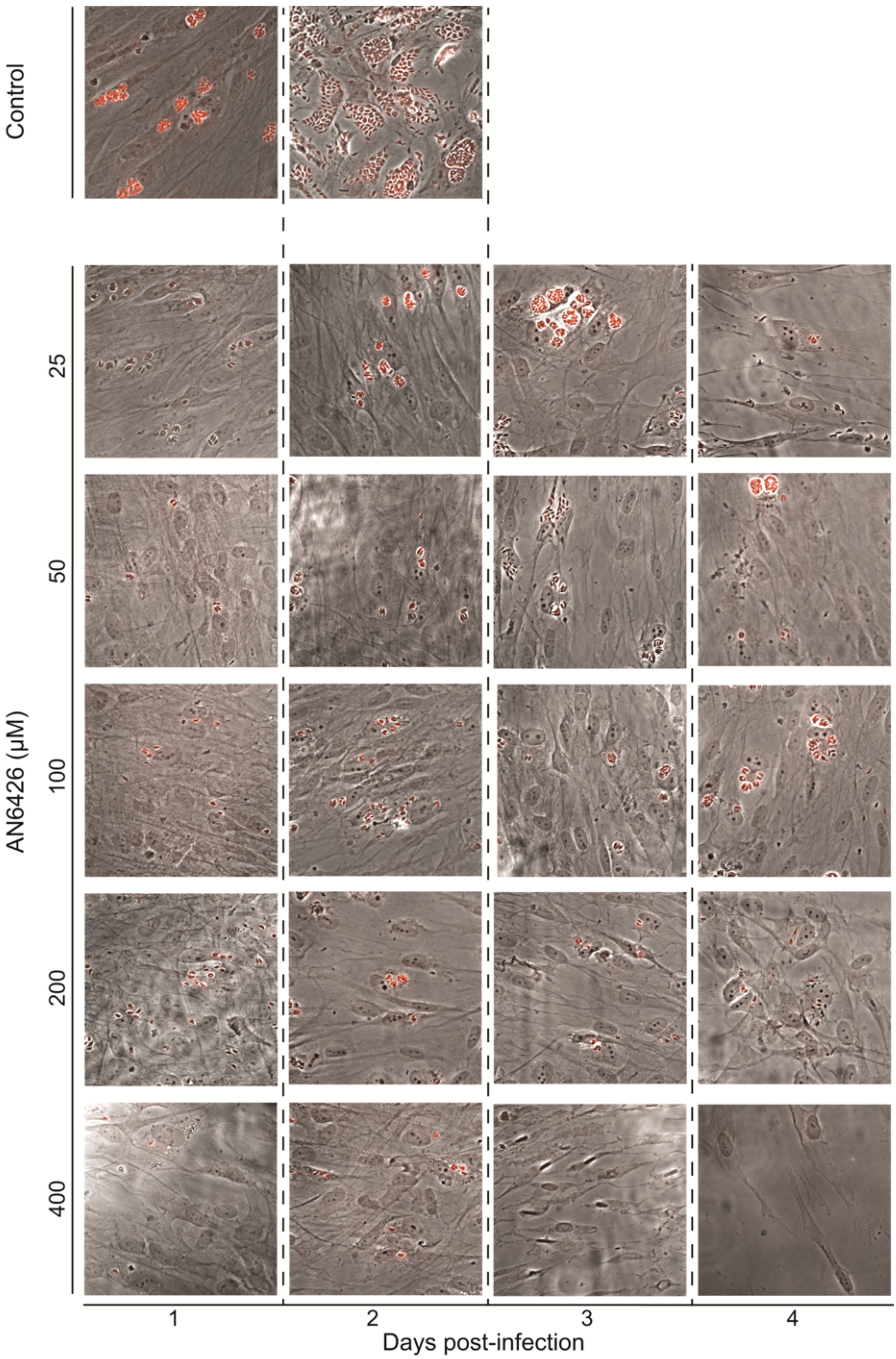

Control
AN6426 (μM)
25
50
100
200
400
1
2
3
4
Days post-infection
